# Stromal Cells Promote Matrix Deposition, Remodelling and an Immunosuppressive Tumour Microenvironment in a 3D Model of Colon Cancer

**DOI:** 10.3390/cancers13235998

**Published:** 2021-11-29

**Authors:** Niamh A. Leonard, Eileen Reidy, Kerry Thompson, Emma McDermott, Eleonora Peerani, Elena Tomas Bort, Frances R. Balkwill, Daniela Loessner, Aideen E. Ryan

**Affiliations:** 1 Lambe Institute for Translational Research, School of Medicine, College of Medicine, Nursing and Health Sciences, National University of Ireland Galway, H91 V4AY Galway, Ireland; e.reidy3@nuigalway.ie; 2 Regenerative Medicine Institute (REMEDI), School of Medicine, College of Medicine, Nursing and Health Sciences, National University of Ireland Galway, H91 W2TY Galway, Ireland; 3 Discipline of Pharmacology and Therapeutics, School of Medicine, College of Medicine, Nursing and Health Sciences, National University of Ireland Galway, H91 W2TY Galway, Ireland; 4 Centre for Microscopy and Imaging, Anatomy, School of Medicine, National University of Ireland Galway, H91 W2TY Galway, Ireland; kerry.thompson@nuigalway.ie (K.T.); emma.mcdermott@nuigalway.ie (E.M.); 5 Barts Cancer Institute, Queen Mary University of London, London EC1M 6BQ, UK; e.f.peerani@qmul.ac.uk (E.P.); e.tomasbort@qmul.ac.uk (E.T.B.); f.balkwill@qmul.ac.uk (F.R.B.); daniela.loessner@monash.edu (D.L.); 6 Faculty of Engineering and Faculty of Medicine, Nursing and Health Sciences, Monash University, Melbourne, VIC 3800, Australia; 7 Leibniz-Institut für Polymerforschung Dresden e.V., 01069 Dresden, Germany

**Keywords:** colorectal cancer, tumour microenvironment, hydrogels, mesenchymal cells, extracellular matrix, 3D model, immune, inflammation, stroma

## Abstract

**Simple Summary:**

Colorectal cancer is the third most common type of cancer in the world. Immune cells and normal supporting cells (MSCs) within a tumour affect patient survival and change how well treatments work. This research aimed to develop a more relevant 3D cancer model that combines MSCs and immune cells with cancer cells to test the effects of multiple cell types on tumour growth. We successfully developed a 3D model that shows that MSCs and immune cells can change the cancer-supporting environment around the tumour cells. We show that combining MSCs and immune cells with cancer cells can increase the level of immune-suppressing molecules they release and change immunotherapeutic drug targets on the cancer cells, similar to changes seen in human tumours. Using this 3D model for research may be better for testing new drugs than traditional 2D methods and could enable the identification of new drug targets.

**Abstract:**

Colorectal cancer (CRC) is the third leading cause of cancer-related deaths worldwide. CRC develops in a complex tumour microenvironment (TME) with both mesenchymal stromal cells (MSCs) and immune infiltrate, shown to alter disease progression and treatment response. We hypothesised that an accessible, affordable model of CRC that combines multiple cell types will improve research translation to the clinic and enable the identification of novel therapeutic targets. A viable gelatine-methacrloyl-based hydrogel culture system that incorporates CRC cells with MSCs and a monocyte cell line was developed. Gels were analysed on day 10 by PCR, cytokine array, microscopy and flow cytometry. The addition of stromal cells increased transcription of matrix remodelling proteins FN1 and MMP9, induced release of tumour-promoting immune molecules MIF, Serpin E1, CXCL1, IL-8 and CXCL12 and altered cancer cell expression of immunotherapeutic targets EGFR, CD47 and PD-L1. Treatment with PD153035, an EGFR inhibitor, revealed altered CRC expression of PD-L1 but only in gels lacking MSCs. We established a viable 3D model of CRC that combined cancer cells, MSCs and monocytic cells that can be used to research the role the stroma plays in the TME, identify novel therapeutic targets and improve the transitional efficacy of therapies.

## 1. Introduction

Colorectal cancer (CRC) is the third most common cancer worldwide and remains a significant health challenge, with over 1.8 million people diagnosed annually [1]. The high mortality rates in CRC are a consequence of the high proportion of CRC patients that present with advanced stage disease [2,3]. Treatments currently used for advanced CRC, including surgery and cytotoxic chemotherapy regimens, show poor efficacy, and mortality in patients with advanced disease remains high [4]. The increase in occurrence rates of CRC has been linked to greater risk in obese and diabetic patients. Despite improvements in screening and limited new therapies, mortality rates have not changed, making CRC the third leading cause of cancer-related deaths [5].

CRC develops in a complex tumour microenvironment (TME), where cancer cells interact dynamically with multiple stromal cell types. In advanced CRC, the stroma accounts for up to 50% of the primary tumour mass [6], with the majority of stromal cells classified as mesenchymal stromal cells (MSCs). MSCs encompass a variety of cell types of mesenchymal origin with similar cell phenotypes, such as myo-fibroblasts and cancer-associated fibroblasts (CAFs), and they can be resident cells or recruited to the tumour site to promote progression [7,8]. Immune cells, both adaptive and innate, are also found in the CRC TME, and depending on their phenotype and location, have different effects on tumour growth and development [9,10,11]. Classification of CRC tumours based on their stromal signature may help predict treatment response and disease-free survival rates [12].

Consensus molecular subtypes (CMS) of CRC identifies four distinct groupings of tumours, CMS1 microsatellite instability immune, CMS2 canonical, CMS3 metabolic and CMS4 mesenchymal [13]. CMS4 tumours compose 23% of all CRC and are characterised by a strong mesenchymal stromal infiltrate, a wound-healing response and matrix remodelling [14]. Mesenchymal cell signatures together with tumour-promoting macrophages are strongly associated with disease progression, advanced pathological stages and worse overall prognosis in CRC [11,15]. Tumour-associated macrophages in CRC have a distinct phenotype compared to macrophages in normal colonic tissue, and changes in macrophage markers such as CD206 and CD163 are associated with decreased overall survival [16,17]. While macrophages have been linked to CRC promotion, they also have anti-tumour potential and are being investigated as a target for novel immunotherapeutics [18,19]. Although evidence exists showing that stromal cell cues shape macrophage infiltration and phenotype, the consequences of these interactions on the extracellular microenvironment and immunological cues in the CRC TME are not well-defined. A better understanding of these interactions may reveal novel therapeutic targets and aid stratification of patients for improved treatment response.

The extracellular matrix (ECM) in CRC supports tumour growth and metastasis, as well as having a protective role in response to a number of treatments [20]. Many systemic therapies, such as chemotherapies and immunotherapies, are more efficacious in liquid tumours than solid, and this is in part due to the infiltration of stromal cells in the TME and deposition of ECM proteins in solid tumours [21,22,23]. Both MSCs and immune cells have been implicated in the deposition and remodelling of the ECM and have been shown to contribute to treatment resistance [15,24,25]. New therapies that alter the ECM are in clinical studies in combination with systemic therapies to improve their efficacy. Furthermore, models that combine key TME compartments that are involved in ECM deposition and remodelling are necessary for identification of targets and testing of treatments in a more physiologically relevant setting.

Immunotherapies have offered hope for new treatment options in a number of cancers in recent years, however, identifying novel cellular and immunological targets in CRC is challenging. The lack of predictability in drug responses in vivo and in clinical trials has resulted in a limited number of novel therapies that have received clinical approval. A variety of in vitro 3D models are used to study cell–cell interactions in CRC as they have considerable advantages over traditional 2D cultures which do not mimic the cell–cell interactions seen in patient tumours [26]. Nonetheless, they often fail to incorporate components of the immune system or MSCs which have dramatic effects on immune cell function and therefore affect immunotherapy response [27]. While animal models better replicate the complexity of the TME, they have species differences that are significant when incorporating human cells to assess immune activation and cell death. In addition, using syngeneic animal models poses a translational problem when antigens and receptors differ between species. This is particularly important when studying immunotherapies and immune cell interactions that rely on complex receptor interactions and signalling cascades [28]. Physiologically relevant models that incorporate multiple cell types may overcome some of the limitations that challenge the field of drug development in CRC research.

Developments in 3D approaches to study CRC have focused on the interactions between multicellular organoids and stromal cell types present in the TME [29]. However, these 3D approaches are limited in the number of cell types that can be incorporated and lack control over the biomechanical properties [30,31]. Moreover, they are difficult to use, costly, have low success rates and are limited by low-throughput screens requiring specialised equipment. To address these limitations, in this study, we used gelatine-methacryloyl (GelMA) hydrogels to mimic multiple elements of the TME in order to understand how different cell populations interact both with the cancer cells and other stromal cells [32]. A better understanding of the cellular interactions in the TME of CRC could lead to the identification of new therapeutic targets and the introduction of novel therapies for patients diagnosed with this disease.

Here, we established a 3D GelMA triple culture system that combines colon cancer cells with MSCs and monocytic THP1 cells. Our 3D model is highly reproducible and supports the viability of multiple cell types within 3D GelMA hydrogels. Our 3D approach facilitated spheroid formation and cellular interactions, ECM deposition and immunomodulatory molecule secretion. Importantly, we detected changes on EpCAM^+^ colorectal cancer cells following co-culture with both MSCs and immune cells. The addition of stromal cells to the culture system altered the cancer’s response to the EGFR tyrosine kinase inhibitor, PD153035. Stromal cells enhanced ECM deposition and immunomodulatory cytokine secretion and the expression of immunotherapeutic targets on CRC cells, validating its use as a novel 3D screening model.

## 2. Materials and Methods

### 2.1. CT26 Culture and Generation of Tumour-Conditioned Medium

Mouse colon adenocarcinoma cells, CT26, derived from Balb/c mice, were purchased from the European Collection of Authenticated Cell Cultures (ECACC) and grown in CT26 media, DMEM (Gibco, ThermoFisher Scientific, Waltham, MA, USA), supplemented with 10% foetal bovine serum (FBS; Sigma-Aldrich, Wicklow, Ireland) and 1% penicillin/streptomycin (ThermoFisher Scientific, Waltham, MA, USA). Tumour-cell secretome (TCS) was generated by plating 1 × 10^6^ CT26 cells in a volume of 25 mL medium for 72 h. Then, the TCS was collected, spun at 1000 RCF for 5 min and stored at −80 °C.

### 2.2. Murine MSC Isolation and Culture

Balb/c mice were purchased from Envigo Laboratories (Oxon, UK), housed and maintained following the conditions approved by the Animal Care Research Ethics Committee of the National University of Ireland, Galway (NUIG), under individual and project authorisation licenses from the Health Products Regulatory Authority (HPRA) of Ireland, in a fully accredited animal housing facility. For murine MSC (mMSC) isolation, Balb/c mice were euthanised by CO_2_. The femur and tibia were removed and cleaned of connective tissue, and bone marrow cells were flushed from the bones. Cells were filtered and plated at a density of 1 × 10^6^ cells per T175 flask (Sarstedt, Wexford, Ireland) at 37 °C in normoxia (21% O_2_) in MEM (ThermoFisher Scientific, Waltham, MA, USA) supplemented with 10% FBS and 1% penicillin/streptomycin. Non-adherent cells were removed 24 h later through a medium change. This process was repeated until cells reached confluency. MSCs were characterised according to the criteria set out by the International Society for Cellular Therapy (ISCT) [31].

### 2.3. Human MSC Isolation and Culture

Human MSCs were isolated from the bone marrow from healthy volunteers at Galway University Hospital under an ethically approved protocol (NUIG Research Ethics Committee, Ref: 8 May 2014). Written consent was obtained from the volunteers. Briefly, bone marrow cell suspensions were layered onto a Ficoll density gradient, and the nucleated cell fraction was collected, washed and re-suspended in hMSC culture medium. After 24 h of culture, non-adherent cells were removed, fresh medium was added and individual colonies of fibroblast-like cells expanded. hMSCs were grown in α-MEM supplemented with 10% FBS, 1% penicillin/streptomycin and fibroblast growth factor 2 (FGF2, 1 ng/mL; Peprotech, London, England). MSCs were characterised according to the criteria set out by the International Society for Cellular Therapy (ISCT) [31].

### 2.4. Human Cell Line Culture

Human CRC cell lines HCT116, HT29, SW480 and the human monocyte cell line THP1 were purchased from American Type Culture Collection (ATCC). Cells were grown in human cell line media, DMEM medium supplemented with 10% FBS, 1% l-glutamine and 1% penicillin/streptomycin. All cells were confirmed mycoplasma-negative (MycoAlert, Lonza, Basel, Switzerland), expanded, frozen and used within 15 passages of testing for all subsequent experiments.

### 2.5. mMSC Treatment for RNA-Seq Analysis

Murine Balb/c MSCs were plated at 3.5 × 10^4^ cells/well in a 6-well plate (Sarstedt, Wexford, Ireland). Then, 24 h after seeding, medium was removed and replaced with fresh MSC medium (40%) mixed with fresh CT26 medium or TCM (60%), and 72 h post-treatment, MSCs were collected for RNA-seq analysis. RNA-seq and data analysis was outsourced to Arraystar (Rockville, MD, USA). Protocol detailed in [33].

### 2.6. CRC Patient Data for In Silico Analysis

CRC patient data were obtained from public sources. mRNA expression data from bulk CRC tumours (*n* = 592) from the TCGA PanCancer atlas [34] were analysed using cBioportal [35,36], and Spearman’s correlation coefficients were determined. Gene expression profiles from two independent CRC datasets were accessed through the NCBI GEO2R repository. Series GSE35602 contained microarray data of stroma and epithelial samples from 13 CRC patients and 4 normal tissues [37]. Series GSE39395 contained microarray data from 8 CRC patients, following FACS sorting, to identify epithelial (EpCAM+, CD45-) and stromal (EpCam-, CD45-) components [38].

### 2.7. Preparation of GelMA-Based Hydrogels

GelMA hydrogels were prepared as reported [32]. Briefly, lyophilised GelMA was dissolved at 37 °C in PBS (Invitrogen, ThermoFisher Scientific, Waltham, MA, USA), 5% or 7.5% (wt/vol) GelMA of the final volume, with 90% of final volume PBS and 10% pre-prepared photo-initiator. The photo-initiator, Irgacure 2959 (Sigma-Aldrich, Wicklow, Ireland), was dissolved in PBS to a final concentration of 2.5 mg/mL and heated to 70 °C for 10 min and filtered through a 0.2 μm syringe filter before adding to the GelMA solution. Cells of interest were re-suspended in the GelMA solution: CRC cell monocultures 1.75 × 10^5^ cells/mL, triple culture 1.75 × 10^5^ HCT116 cells/mL + 8.75 × 10^4^ THP1 cells/mL + 8.75 × 10^4^ MSCs/mL or MSC^High^ at 3.5 × 10^5^ cells/mL. Cell-containing GelMA was pipetted in a Teflon casting mould (50 × 4 × 2 mm), covered with a glass slide and placed in a UV cross-linker at 365 nm for 10 min. Then, photo-cross-linked hydrogels were removed from the mould and cut into 4 × 4 × 2 mm hydrogels using a cutting guide. Triple cultures were grown in 50% human cell line medium and 50% human MSC medium, replaced on day 1 and day 8. EGFR tyrosine kinase inhibitor, PD153035 (1 μM), or 0.01% DMSO, was used to treat 3D cell cultures on day 4 and day 7. All readouts were collected on day 10, unless stated otherwise.

### 2.8. Analysis of 3D Cell Cultures

Mechanical testing was conducted on day 10 on cell-containing hydrogels. The cross-sectional area was calculated for each hydrogel and the mechanical properties of the samples were determined by unconfined compression testing with a 10N load cell on an Instron 3342. The Young’s modulus was determined by calculating the slope at the linear region of the stress–strain graph. Cell proliferation and metabolic activity of 3D cultures were determined by CyQuant and AlamarBlue assays respectively, as previously reported [32]. Cell viability of 3D cultures was assessed using a LIVE/DEAD™ Viability/Cytotoxicity Kit (Invitrogen, ThermoFisher Scientific, Waltham, MA, USA), as described earlier [39]. Samples were imaged using the FLUOVIEW FV3000 confocal laser scanning microscope (Olympus), and the percentage of live/dead (Calcein-AM/Propidium iodide (PI)) cells was determined using the FijI ImageJ software.

### 2.9. Scanning Electron Microscopy

To visualise the ultrastructure of cell spheroids, cell-containing hydrogels were digested on day 10 using 0.5 mg/mL of collagenase (C2674-1G, Sigma-Aldrich, Wicklow, Ireland) for 3 h at 37 °C. All subsequent steps were performed in a fume hood. Samples were washed with PBS and fixed in 2% glutaraldehyde and 2% paraformaldehyde in 0.1 M sodium cacodylate buffer, pH 7.2, for 2 h at room temperature. Samples were dehydrated through a graded series of ethanol (30%, 50%, 70%, 90%, 100%) for 2 × 15 min. The spheroids were then placed in hexamethyldisilazane for 2 × 15 min. Following the final HMDS dehydration steps, the spheroids were gently centrifuged, excess HMDS was removed and the spheroids were re-suspended in 200 µL of fresh HMDS. The spheroid suspension was then pipetted onto a glass slide and left to air-dry overnight. The glass slide was then mounted onto an aluminium SEM stub using a double-sided carbon tab and gold sputter-coated (Quorum Q150R ESplus). Samples were imaged on the Hitachi S-2600N scanning electron microscope (Hitachi, Krefeld, Germany).

### 2.10. Transmission Electron Microscopy

All steps were performed in a fume hood. Day 10 gels were placed in 2% glutaraldehyde and 2% paraformaldehyde in 0.1 M sodium cacodylate buffer, pH 7.2, for 2 h at room temperature. Then, samples were placed in secondary fixative (1% osmium tetroxide) for 2 h prior to processing. Samples were dehydrated through a graded series of ethanol (30%, 50%, 70%, 90%, 100%) for 2 × 15 min followed by acetone for 2 × 20 min. The samples were then gradually infiltrated with resin (Agar Low-Viscosity Resin AGR1078) by placing the gels in a 50:50 resin and acetone mixture for 4 h, then a 75:25 resin and acetone mixture overnight and 100% resin for 6 h. Samples were transferred to an embedding mould filled with fresh 100% resin and then polymerised at 65 °C for 48 h. Afterwards, 500 nm semi-thin sections were cut using a glass knife, transferred onto a glass slide, stained with toluidine blue and viewed using a light microscope. From areas of interest, 70–90 nm ultrathin sections were cut using a diamond knife on a Lecia UC6 ultramicrotome, placed on 3 mm copper grids and allowed to air-dry. Sections were stained with UA-Zero EM stain (Agar) for 10 min and imaged on the Hitachi-7500 transmission electron microscope.

### 2.11. Real-Time Quantitative PCR

For gene expression analysis, four cell-containing hydrogels were combined in GentleMACS M tubes (Miltenyi Biotec, Bergisch Gladbach, Germany) with 600 μL of trizol (Sigma-Aldrich, Wicklow, Ireland) and placed in a gentleMACS dissociator. The digest was transferred to a 2 mL tube in the fume hood and 1/5 of the trizol volume of chloroform (Sigma-Aldrich) was added. Samples were agitated and incubated for 5 min before centrifuging at 1.4 × 10^4^ RCF for 15 min at 4 °C. The transparent upper fraction was extracted and added to an equal volume of isopropanol (Sigma-Aldrich, Wicklow, Ireland). Samples were agitated and incubated for 10 min before centrifuging at 1.4 × 10^4^ RCF for 20 min at 4 °C. The supernatant was discarded, the pellet was washed with 80% ethanol (Sigma-Aldrich), then 100% ethanol before air-drying the pellet, which was then re-suspended in 20 μL of RNAse-free water. After DNase I (EN0521, ThermoFisher Scientific, Waltham, MA, USA, EN0521), RNA quality and concentration were determined using a Nanodrop. RNA was converted to cDNA using the RevertAid H Minus First-Strand cDNA Synthesis Kit (K1632, ThermoFisher Scientific, Waltham, MA, USA). RT-qPCR analysis was carried out using the Maxima Probe/ROX qPCR Master Mix (K0231, ThermoFisher Scientific, Waltham, MA, USA) on the Applied Biosystems StepOnePlus Real-Time PCR system. TaqMan™ Gene Expression Assay probes were used with B2M as a house-keeping gene (Table 1).

### 2.12. Human Cytokine Array

The Proteome Profiler Human Cytokine Array Kit (ARY005B, R&D systems, Minneapolis, MN, USA) was used to assess the secretion of cytokines in the 3D cell cultures. On day 8, cell-containing hydrogels were placed in 500 μL of fresh medium per well of a 48-well plate (Sarstedt, Wexford, Ireland) for 48 h. The cell conditioned medium was collected and stored at −80 °C until analysis according to the manufacturer’s recommendations. Briefly, samples and a detection antibody cocktail were incubated with the membrane overnight at 4 °C. The membrane was washed and incubated with Streptavidin-HRP for 30 min, followed by another wash and Chemi-reagent. Signal density was analysed using ImageJ.

### 2.13. Flow Cytometry

Cell-containing hydrogels were digested as above and then spheroids were placed in trypsin for 20 min. The cell suspension was passed through a 70 μm filter and 1 × 10^5^ cells/well were plated. Surface staining for EpCAM, PD-L1, CD47 and EGFR (Table 2) was completed at 4 °C for 15 min. Sytox blue viability dye was added prior to sample acquisition. Intracellular staining for Ki67 required staining with EpCAM and zombie viability dye at 4 °C for 15 min. Samples were washed twice before fixing in 2% PFA (Fixation Buffer, 420801, BioLegend, San Diego, CA, USA) for 20 min at room temperature, protected from light. The samples were washed twice in permeabilization wash buffer (421002, BioLegend, San Diego, CA, USA) before staining with Ki67 (Table 2) for 15 min at room temperature. Samples were run on the Cytek Northern Lights 2000 flow cytometer and analysed using FlowJo version 10. 

### 2.14. Statistical Analysis

Statistical analysis was performed using GraphPad^®^ version 9 (La Jolla, CA, USA). Data were assessed for normal distribution using the D’Agostino–Pearson omnibus normality test. Datasets with two groups were analysed using an unpaired *t* test. Datasets with more than two groups were analysed by ordinary one-way ANOVA followed by the Tukey, Šídák’s or Dunnetts multiple comparison tests. Results were considered statistically significant at *p* < 0.05.

## 3. Results

### 3.1. Tumour Stromal Cells Have Increased Matrix Deposition, Matrix Remodelling and Immune Suppression Signatures Compared to Normal Stroma

Mesenchymal cells in the colon make up a substantial proportion of the cells surrounding the intestinal epithelium. The role the stromal compartment, both immune and mesenchymal, plays in tumour development has become an area of interest in recent years. Moreover, the stromal compartment of the TME is a prognostic indicator of disease-free survival [12]. Patients with strong tumour mesenchymal stromal signatures have the lowest disease-free survival rates [13]. The mechanism by which the stromal compartment affects tumour progression and response to therapies is not fully understood. Targeting ECM and tumour-promoting inflammation have been areas of interest for the development of novel therapies for the treatment of CRC as they have been shown to promote tumour growth and metastasis and reduce survival [20,40]. However, the precise functional roles of the stroma in supporting and shaping the tumour microenvironment are not well-understood.

**Figure 1:**
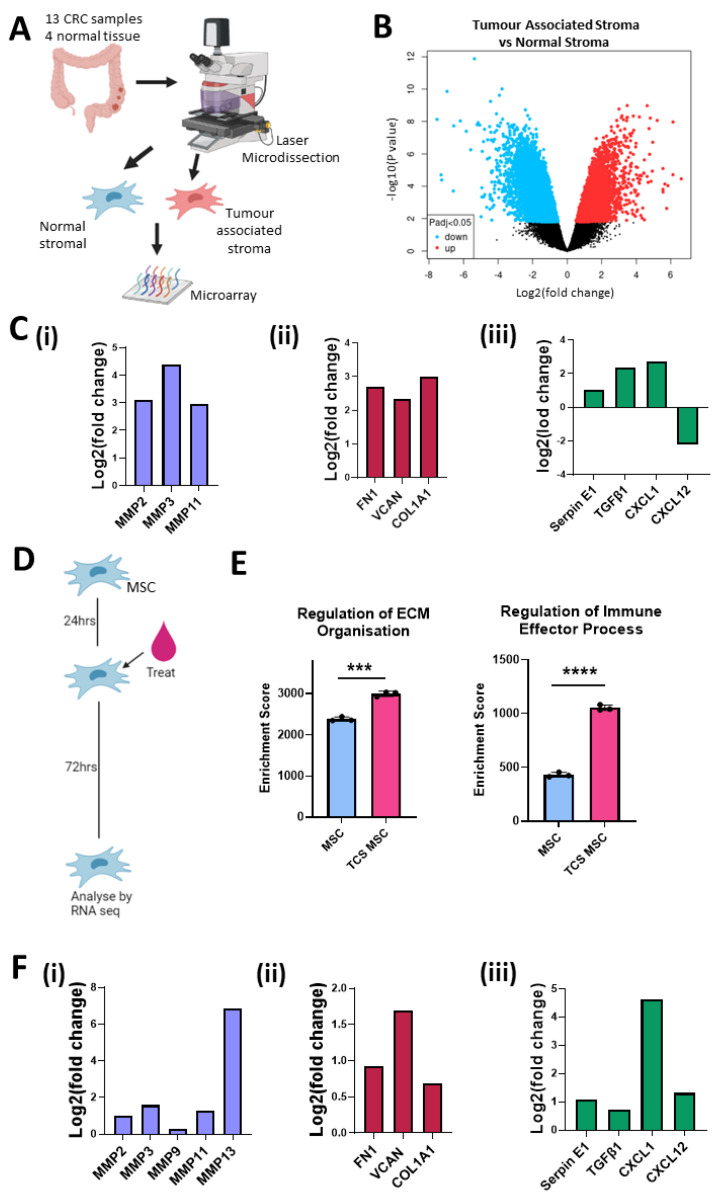
Stromal cells in the colon tumour microenvironment are altered and increase expression of matrix remodelling, ECM and immunomodulation transcripts. (**A**) Schematic description of how samples from GSE35602 were processed. (**B**) Volcano plot of tumour-associated stroma (*n* = 13) versus normal stroma (*n* = 4) differential mRNA expression (GSE35602). (**C**) Fold change of mRNA levels related to matrix remodelling (MMP2, MMP3, MMP11) (**i**), ECM deposition (FN1, VCAN, COL1a1, COL11A1) (**ii**) and immune modulation (Serpin E1, TGFβ1, CXCL1, CXCL12) (**iii**) in tumour-associated stroma versus normal stroma (GSE35602). (**D**) Schematic description of treatment timeline for mMSC TCS conditioning. (**E**) Gene set enrichment analysis results for control mMSC (blue) (*n* = 3) and TCM mMSC (Pink) (*n* = 3) RNAseq. (**F**) Fold change of mRNA levels related to matrix remodelling (MMP2, MMP3, MMP9, MMP11, MMP13) (**i**), ECM deposition (FN1, VCAN, COL1a1) (**ii**) and immune modulation (Serpin E1, TGFβ1, CXCL1, CXCL12) (**iii**) in normal mMSC versus TCS mMSC. Error bars represent mean ± SD. *** *p* < 0.001, **** *p* < 0.0001 by *t* test.

In order to determine the role of the stromal compartment of the TME of CRC, we compared mRNA (GSE35602) from CRC-associated stroma with normal colon stroma by microarray analysis (Figure 1A) and found a large number of differentially regulated genes (Figure 1B). Our results display how CRC can act on the surrounding stromal cells and alter their transcriptome, with the capacity to modify their phenotype. The tumour-associated stromal cells showed a significant increase in mRNA encoding ECM proteins (Figure 1C(ii)), MMPs which play a vital role in matrix reorganisation (Figure 1C(i)) and immunomodulatory molecules (Figure 1C(iii)). The mRNA levels of FN1, VCAN and COL1A1, all ECM proteins found in the TME of CRC, are implicated in tumour growth, metastasis and poor prognosis, and were significantly increased in the CRC-associated stroma (Figure 1C(ii)) [41,42,43]. We found a significant increase in MMP2, MMP3 and MMP11, all proteinases that remodel the ECM, acting on gelatine (MMP2, MMP9) or collagen (MMP11, MMP13), in the CRC-associated stroma (Figure 1C(i)) [44]. The increased expression of these ECM-remodelling enzymes in the TME of CRC has been linked to survival outcomes and metastasis [45,46,47]. The immunomodulatory factors SerpinE1, TGFβ1, CXCL1 and CXCL12 act directly on immune cells to induce a wound-healing phenotype that contributes to angiogenesis, epithelial-to-mesenchymal transition (EMT), tumour growth and metastasis. The expression of these tumour-supporting immune molecules was significantly increased in the CRC-associated stroma (Figure 1C(iii)) [48,49,50,51].

To validate the effects seen in the stromal compartment, we conducted RNAseq analysis of mMSCs conditioned with tumour cell secretome (TCS) (Figure 1D). Gene set enrichment analysis revealed that TCS-treated MSCs had higher enrichment scores for transcription pathways involved in the regulation of ECM organisation and regulation of immune effector processes compared to MSCs exposed to fresh medium (Figure 1E). When we looked at changes in individual mRNA transcripts, we observed an increase in matrix reorganisation genes (Figure 1F(i)), ECM proteins (Figure 1F(ii)) and immunomodulatory factors (Figure 1F(iii)), supporting the data shown in Figure 1C. Together, these data indicate that CRC cells alter the transcriptional profile of the surrounding stroma, in particular MSCs. Increased mRNA levels were related to ECM organisation and immune modulation in the CRC-associated stroma. These pathways have been implicated in tumour-promoting inflammation, invasion, metastasis and immunosuppression. Changes in the expression of these tumour-supporting elements may enable targeting of tumour-associated stroma to disrupt the tumour-supporting microenvironment while localising the effects to the TME and protecting the adjacent normal stroma.

### 3.2. Higher Levels of Matrix Organisation and Immunomodulation Pathways in Tumour Stromal Cells Compared to Cancer Cells

**Figure 2:**
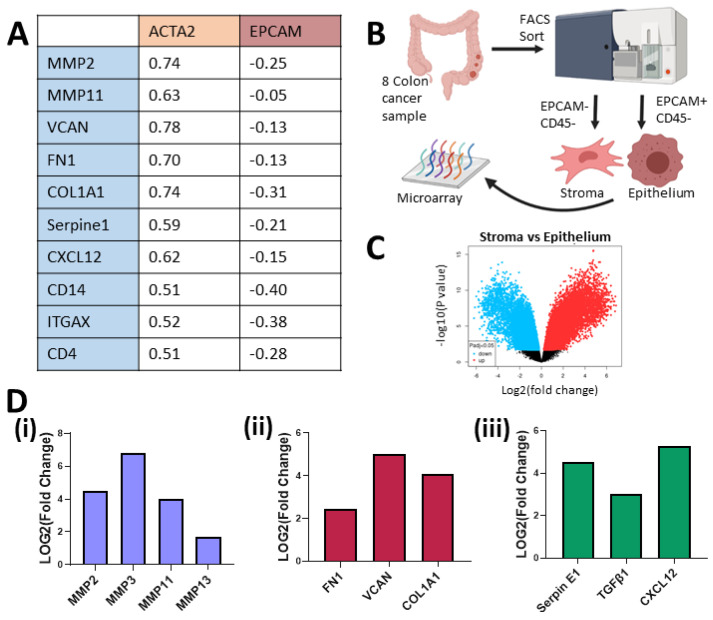
Stromal cells in the TME express higher mRNA levels than the tumour cells for a number of matrix remodelling, ECM and immunomodulation genes. (**A**) Spearman correlation from mRNA of bulk colorectal adenocarcinoma, TCGA, PanCancer Atlas, cBioportal (*n* = 592). (**B**) Schematic description of how samples from GSE39397 were processed. (**C**) Volcano plot of stroma (−EpCAM, −CD45) versus epithelium (+EpCAM, −CD45) from colon cancer tumours’ (*n* = 8) differential mRNA expression (GSE39397). (**D**) Fold change of mRNA levels related to matrix remodelling (MMP2, MMP3, MMP9, MMP11, MMP13) (**i**), ECM deposition (FN1, VCAN, COL1A1) (**ii**) and immune modulation (Serpin E1, TGFβ1, CXCL12) (**iii**) in tumour-associated stroma versus cancer epithelium (GSE39397).

Following the identification of tumour-promoting transcripts induced in the CRC-associated MSCs, we next investigated the impact of these transcripts in the TME compared to CRC cells. Analysis of bulk tumour mRNA displayed a strong correlation between ACTA2, a marker of MSCs, and matrix remodelling enzymes (MMP2), ECM proteins (VCAN, FN1, COL1A1), immunomodulatory molecules (Serpine1, CXCL12), innate immune cell markers (CD14, ITGAX) and adaptive immune cell markers (CD4) (Figure 2A). When we compared the correlation between the aforementioned genes and EpCAM, an epithelial cell marker, we observed a weak negative correlation (Figure 2A), suggesting that these tumour-promoting factors may be associated with the mesenchymal compartment of the TME rather than the CRC cells. To further investigate this, we analysed expression levels from a dataset (GSE39397) that FACS-sorted CRC tumours (Figure 2B). We found that CRC vastly altered the mRNA of the tumour-associated stromal cells in Figure 1, while here we show that EpCAM+ CRC cells still possessed a very distinct transcriptome to the stroma with an array of differentially regulated transcripts (Figure 2C). The mRNA levels that were significantly upregulated in the stromal compartment when compared to cancer compartment include MMP2, MMP3, MMP11 and MMP13, all involved in tissue remodelling and cell invasion (Figure 2D(i)), the ECM genes FN1, VCAN, and COL1A1, all associated with poor prognosis (Figure 2D(ii)), and chemokines and cytokines, Serpin E1, TGFβ1 and CXCL12, which alter immune cell function and promote tumour growth (Figure 2D(iii)). These data suggest that the stromal compartment may play a significant role in the regulation of processes in tumour initiation and progression as well as immunomodulation. While this data from bulk tumour and traditional 2D culture systems can provide significant insight into changes in the TME, it can be difficult to study individual signalling pathways between the different cell types in a controlled manner.

### 3.3. GelMA Supports Incorporation of Multiple Cell Types, Including Stromal Cells and Macrophages, and Facilitates CRC Proliferation

**Figure 3:**
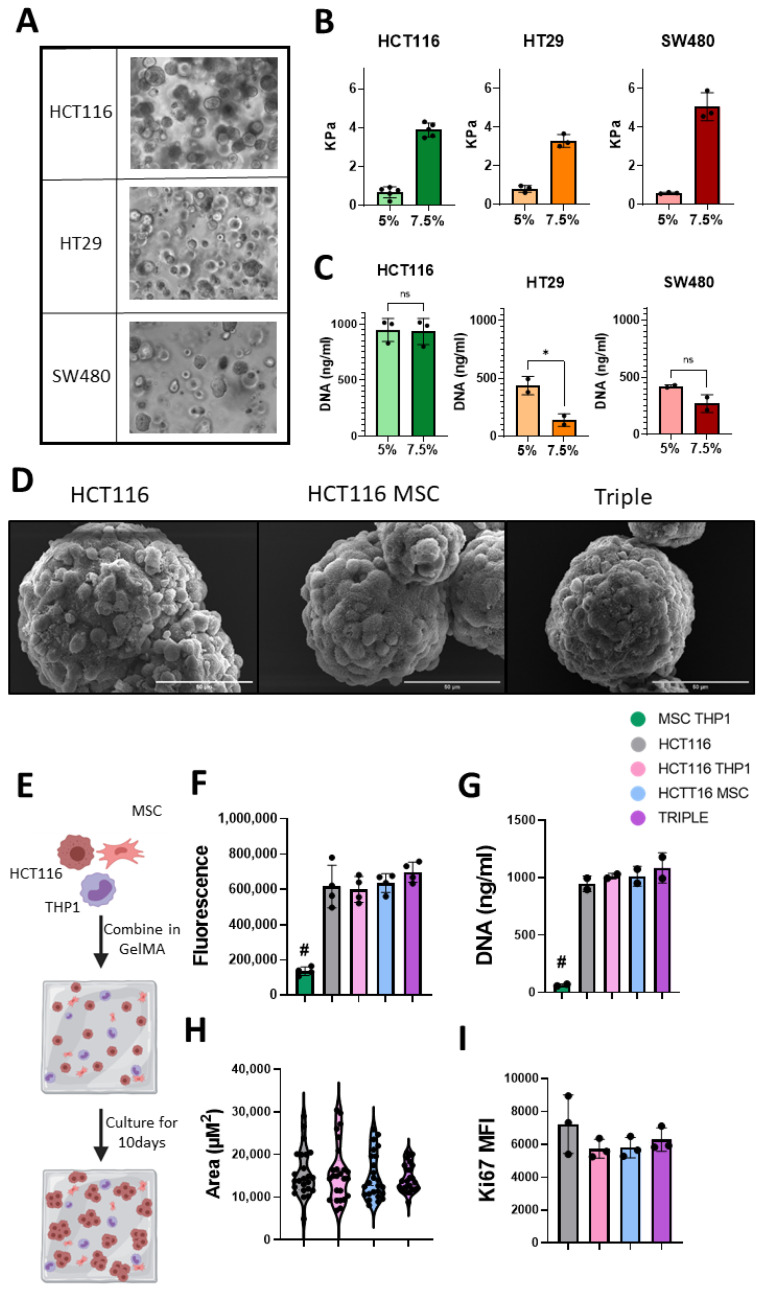
GelMA culture system supports the growth of colon cancer cell lines within triple culture system. (**A**) Light microscopy images of spheroid formation from 3 CRC cell lines, HCT116, HT29 and SW480, at day 10 culture in 5% GelMA hydrogel, 20× magnification. (**B**) Rheometry measurements of GelMA stiffness at day 10 of culture (*n* = 3–5). (**C**) Proliferation of CRC cells at day 10 in 5% and 7.5% gels (*n* = 2–3) measured by CyQuant DNA analysis. (**D**) Representative SEM images of spheroid isolated from gels at day 10, scale = 50 μm. (**E**) Schematic description of 10-day GelMA triple culture system. (**F**) Alamar Blue measure of metabolic activity of cell contain gels at day 10 (*n* = 4). (**G**) Proliferation of cells at day 10 (*n* = 2) measured by CyQuant DNA analysis. (**H**) Spheroid size at day 10 (biological *n* = 5, technical *n* = 5). (**I**) Flow cytometry Ki67 expression in EpCAM positive cells isolated at day 10 (*n* = 3). Individual values are displayed as black dots on bar charts. Error bars represent mean ± SD. * *p* < 0.05, # *p* < 0.005 compared to all other groups, by *t* test or one-way ANOVA and Tukey post hoc test.

3D models are being used in tumour biology more frequently as they allow the formation of tumour spheroids, cell–cell and cell–matrix interactions, deposition of ECM molecules and the controlled addition of multiple cell types [26]. GelMA-based hydrogels were employed as they contain binding sites to promote cell adhesion and growth, while also facilitating the modulation of the mechanical properties [32]. As CRC tissue increases in stiffness as the disease stage progresses [52], three CRC cell lines, HCT116, HT29 and SW480, were incorporated in 5% and 7.5% (*w*/*v*) hydrogels. Following unconfined compression testing, hydrogels with 5% GelMA resulted in softer hydrogels than 7.5% GelMA gels (Figure 3B). The hydrogel-based 3D cultures supported spheroid formation of the three CRC cell lines with the largest spheroids observed in the HCT116 containing 5% GelMA hydrogels (Figure 3A). In addition, by day 10 of 3D culture, HCT116 cells proliferated to a greater extent than the HT29 and SW480 cells (Figure 3C). HT29 cells proliferated significantly more in the 5% GelMA hydrogel, whereas the HCT116 and SW480 cells grew equally well in both hydrogel conditions (Figure 3C).

In addition to the HCT116 cells proliferating at a higher rate in both hydrogel conditions, they are a CMS4-like cell line with an mRNA signature similar to that seen in mesenchymal-dense tumours with an immune infiltrate. CMS4 tumours have the lowest disease-free survival rate [53]. HCT116 cells are a microsatellite instable cell line, making them a good model for investigating immunogenic cell death. They also display strong invasive behaviour which is important for studying metastasis [54,55]. For these reasons, we chose HCT116 cells and 5% hydrogels to incorporate mesenchymal stromal cells and monocytic THP1 cells to mimic monocyte/macrophages in the TME (Figure 3E). Scanning electron micrographs indicated well-formed spheroids isolated from the hydrogel-based 3D cultures on day 10 (Figure 3D). Spheroids in the 3D triple cultures appeared more compact and had a more uniform surface than HCT116 monocultures (Figure 3D).

The integrity of cellular and organelle structures and orientation in the spheroids was assessed by transmission electron microscopy (TEM) (Supplementary Figure S1). The spheroids displayed a characteristic epithelial cell shape, which is representative of their in vivo architecture but often lacking when grown in 2D, with cells on the outside of the spheroid being polarised in a similar manner as seen in the gut lining. In addition to large nuclei, which is a characteristic of cancer cells, intact cellular components, such as the endoplasmic reticulum, Golgi and cytoskeletal filaments, were observed (Supplementary Figure S1). The cells displayed a high number of mitochondria, suggesting high metabolic activity. Desmosomes, cell–cell connections which infer stability, were visible between the CRC cells, but few other cell junctions were visible. A reduction of cell junctions could be attributed to EMT and metastasis [56,57]. However, there were numerus sites of endocytosis and exocytosis, suggesting protein trafficking that may facilitate a tumour-promoting TME (Supplementary Figure S1(iv)) [58].

The nucleus size and number of mitochondria did not change between the different 3D cell cultures. In the 3D triple cultures, interdigitations between CRC cells were abundant, which have previously been shown as a characteristic of EMT. Autolysosomes, which are implicated in cancer metastasis, increased in the 3D triple cultures compared to the HCT116 monocultures (Supplementary Figure S1) [58]. Together, these finding suggest that the addition of MSCs and monocytes to the 3D CRC cell cultures promoted characteristics associated with EMT and metastasis. These characteristics are hallmarks of late-stage disease, highlighting the relevance of our 3D model for developing and testing new anti-cancer therapeutics in advanced-stage CRC.

### 3.4. GelMA Supports Viability in Multicellular 3D Cultures

MSCs and monocytes influence cancer progression, but the interactions that underpin this process have yet to be elucidated and targeted therapeutically. Thus, we sought to determine the behaviour of CRC cells upon addition of MSCs and THP1 cells in our 3D model and determined the metabolic activity. 3D stromal cell cultures (MSCs + THP1) did not display a high metabolic activity, and when added to the cancer cells, their metabolic rate was unchanged (Figure 3F). Determining the proliferation of the 3D stromal cell cultures using a DNA-based assay (Figure 3G) and the proliferation marker Ki67 by flow cytometry (Figure 3I) at day 10, we observed the same result. Analysing the spheroid size (Figure 3H), we found no significant difference between the average spheroid size, although the spheroids in the 3D triple cultures displayed a more uniform size (Triple SD = 3295 μm^2^) compared to the control groups (HCT116 SD = 5604 μm^2^, HCT116 + THP1 SD = 6862 μm^2^, HCT116 + MSC SD = 5378 μm^2^).

**Figure 4:**
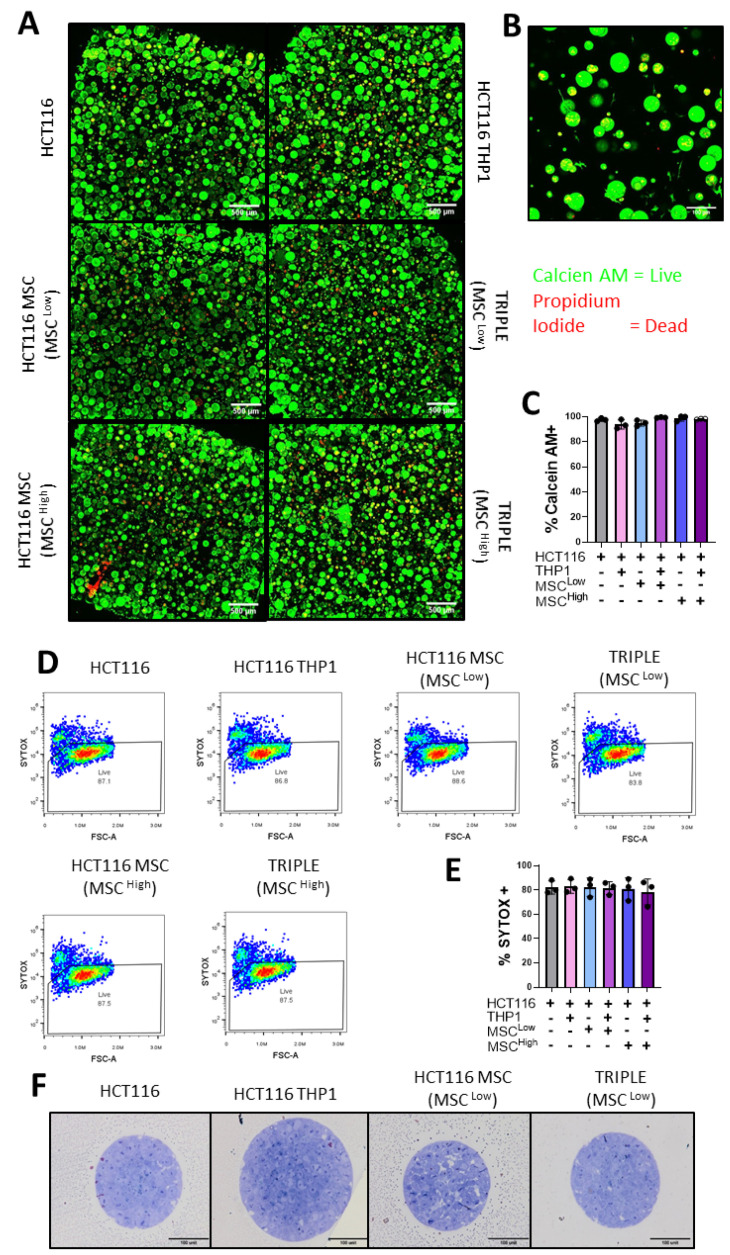
Epithelial, mesenchymal and immune cells are viable in the 3D model of colon cancer. (**A**) Confocal microscopy images of Calcein AM- (live) and Propidium Iodide (dead)-stained gels, 4× magnification. (**B**) Representative confocal image of Calcein AM- (live) and Propidium Iodide (dead)-stained Triple (MSC^high^) gel, 20× magnification. (**C**) Quantification of % of Calcein AM-positive or live cells in the gels (*n* = 3 fields within 1 gel). (**D**) Representative Sytox Blue flow cytometry plots for viability analysis. (**E**) Quantification of flow cytometry Sytox Blue viability stain on dissociated gels (*n* = 3). (**F**) Light microscope images of semi-thin resin cross-sections from the spheroids stained with toluidine blue. Individual values are displayed as black dots on bar charts. Error bars represent mean ± SD. One-way ANOVA and Tukey post hoc test.

The viability of the tumour spheroids, primary MSCs and monocytes was an important consideration when developing our 3D CRC model. Hence, we performed live/dead imaging that captured almost the full field of view for the 3D cultures (Figure 4A). There was no cell death apparent in the centre of the cell-containing hydrogels, suggesting good perfusion of nutrients throughout the hydrogel. Upon examination of a higher magnification of the 3D triple (MSC^high^) cultures, we found larger spheroids, indicative of HCT116 cell growth, and elongated cells characteristic of primary MSCs (Figure 4B). The spheroid viability was assessed by quantifying the staining intensity, yielding over 90% viability in each 3D culture condition (Figure 4C). We also observed intact cellular structures within the spheroids in addition to the external layer, indicating that viability is maintained throughout the spheroids (Figure 4F).

To validate these results, we conducted flow cytometry analysis. Following the digestion of the hydrogel, dissociation of the spheroids and cell staining, we measured an average of 80% viability in each 3D culture condition (Figure 4D,E). Taken together, our results show that the GelMA 3D platform supports the growth of primary MSCs and immune cells, which can be very sensitive to their culture system, while also allowing the formation and proliferation of tumour spheroids. This supports the use of the model in CRC research.

### 3.5. Upregulation of Genes Related to the ECM and Matrix Reorganisation Following Incorporation of Tumour Stromal Cells in GelMA

**Figure 5:**
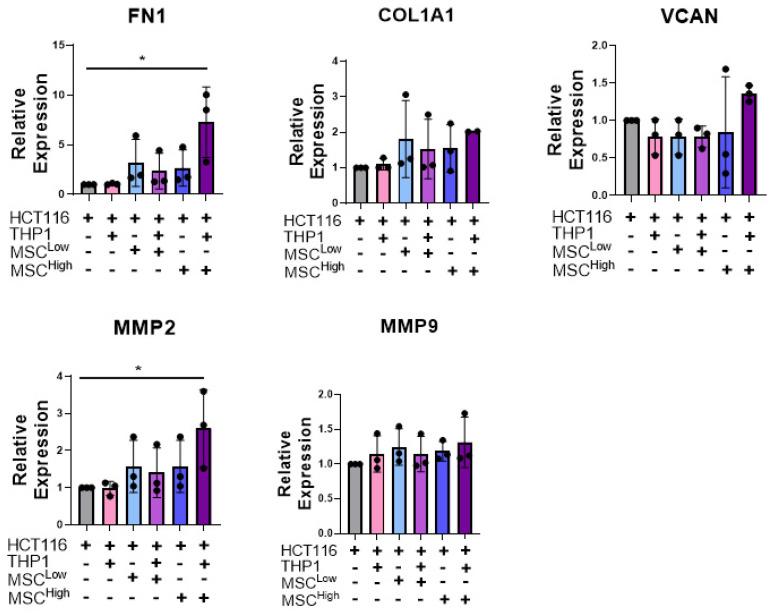
Stromal cells increase mRNA levels of ECM genes and matrix remodelling genes in a triple culture. RT-qPCR analysis of mRNA levels of the ECM components and matric remodelling enzymes FN1, COL1A1, VCAN, MMP2 and MMP9 in whole gels at day 10. Individual biological replicates are displayed as black dots on bar charts. Error bars represent mean ± SD. * *p* < 0.05. One-way ANOVA and Dunnetts multiple comparison test (HCT116 compared to all other groups).

We next assessed the role of stromal cells in altering aspects of ECM organisation, which affects treatment response and promotes invasion. The expression of ECM proteins such as Fibronectin 1 (FN1) and collagen type 1 α 1 (COL1A1) have been implicated in promoting migration and invasion in colon cancer [59,60]. The remodelling of these ECM components has been shown to contribute to invasion and metastasis, while also acting in certain tumours as a physical barrier to block immune cell infiltration [23,61]. Given this, we measured mRNA levels of FN1, COL1A1 and versican (VCAN), which are ECM molecules, and MMP2 and MMP9, which are matrix remodelling enzymes (Figure 5).

A significant increase in FN1 mRNA expression was observed in the triple culture (MSC^high^) compared to the cancer cells alone or cancer cells embedded with THP-1 (Figure 5). Similarly, although not significant, there is a trend toward an increase in COL1A1 and VCAN expression in the triple culture (MSC^high^) compared to 3D CRC cell monocultures (Figure 5). We observed a significant upregulation of MMP2 transcripts when MSC^High^ and THP1s were added to the cancer cells compared to the cancer cells cultured alone. These results suggest that the addition of stroma, with a preference for high-density mesenchymal stroma, contributes to or induces the transcription of ECM genes and matrix remodelling genes that have been shown to promote tumour progression.

### 3.6. Stromal Cells Alter the Secretion of Immune Molecules Associated with Tumour Promotion

**Figure 6:**
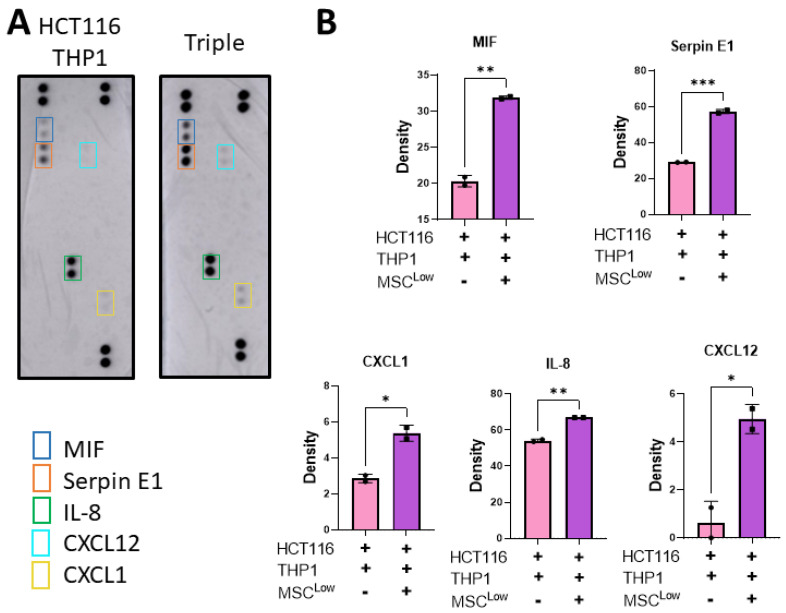
Stromal cells increase the release of tumour-promoting immunomodulatory molecules. (**A**) Gels were placed in fresh media on day 8 and left for 48 h before collection on day 10. The conditioned media was placed on an R&D Proteome Profiler Human Cytokine Array Kit membrane. (**B**) Pixel density of spots on cytokine array membrane for MIF, SerpinE1, CXCL1, IL-8 and CXCL12. Individual values are displayed as black dots on bar charts. Error bars represent mean ± SD. * *p* < 0.05, ** *p* < 0.01, *** *p* < 0.001, by *t* test.

The tumour immune environment can affect tumour progression and response to therapeutics. As the target of emerging therapeutics, macrophages have the capacity to function as either pro-tumour or anti-tumour effectors, which is dictated by the cues in their microenvironment. The addition of MSCs to the culture system (HCT116 and THP1s) increased the release of CXCL1 (Neutrophil-Activating Protein 3), CXCL12 (stromal cell-derived factor 1 α), IL-8 (CXCL8), MIF (macrophage migration inhibitory factor) and SerpinE1 (Plasminogen Activator Inhibitor 1) (Figure 6A,B).

CXCL1, IL-8, CXCL12 and Serpin E1 have an immune modulation function, acting as chemokines to recruit immune cells to the TME or acting on immune cells to alter their function. They can also act directly on cancer cells to promote remodelling of the TME, metastasis and tumour growth [50,61]. The addition of MSCs to the culture system altered the secretome of the 3D model, inducing a tumour-supportive, immune-suppressive TME. This significant alteration in the secretome following the addition of the MSCs displays the potential impact they have in supporting tumour growth and how the use of this model could enable the identification of novel therapeutic targets to patients with stromal-dense tumours.

### 3.7. Stromal Cells Alter Expression of Immunotherapeutic Targets on Cancer Cells

**Figure 7:**
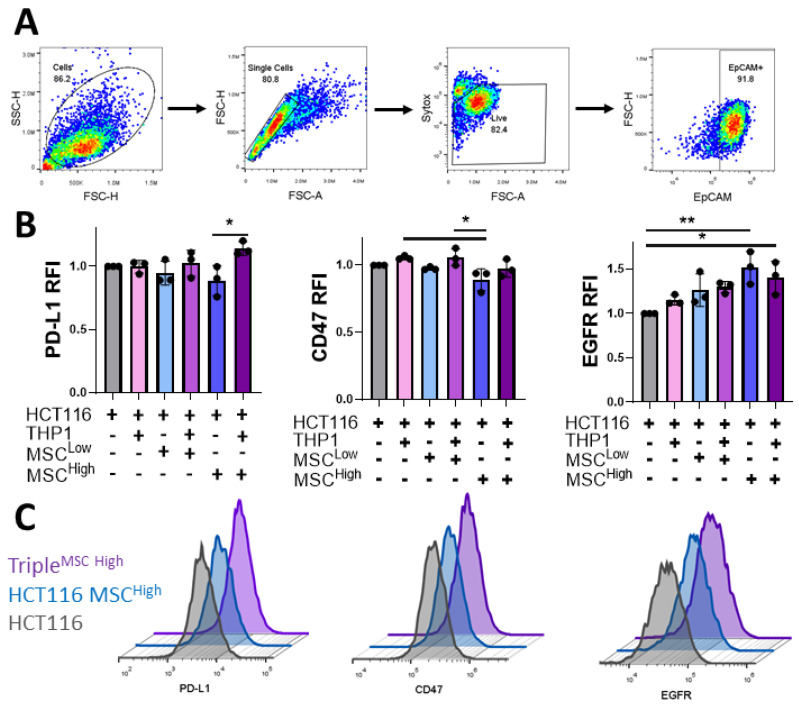
Incorporation of stromal and immune cells in the 3D GelMA model alters cancer expression of immunotherapeutic targets. (**A**) The flow cytometry gating strategy was used to assess cancer cells’ expression from gels dissociated at day 10. Cell debris is gated out initially, followed by gating on single cells, live cells and then the EpCAM+ cancer cells. (**B**) Relative median fluorescent intensity (RFI) of PD-L1, CD47 and EGFR on EpCAM-positive cells isolated from the gels. (**C**) Representative histograms of the flow cytometer staining, HCT116 (grey), triple culture (MSC^Low^) (blue) and triple culture (MSC^High^) (Purple). Individual biological replicates are displayed as black dots on bar charts. Error bars represent mean ± SD. * *p* < 0.05, ** *p* < 0.01, one-way ANOVA and Tukey post hoc test.

We have shown that MSCs can alter the immunomodulatory secretome from the culture system, but the effect on cancer cell expression of immunosuppressive molecules and targets of immunotherapies remains unknown. Better understanding of this may lead to stratification of patients for treatments and identification of novel therapeutics that may work in subsets of patients with stromal-dense tumours. To investigate this, flow cytometry analysis was conducted on live EpCAM+ cells dissociated from the culture system (Figure 7A). PD-L1 binds to its receptor on a variety of immune cells, inhibiting their anti-tumour activity, and this signalling axis has been a target for cancer therapies in recent years [62]. Our data display a trend towards a reduction in HCT116 PD-L1 expression with MCS inclusion in the culture system, which is overcome with the addition of THP1 cells (Figure 7B). PD-L1 expression has been linked to interferon signalling, and the incorporation of an immune cell component such as THP1s may be necessary to induce its expression by promoting an inflammatory TME, an emerging hallmark of cancer [63,64].

A similar trend is seen with the expression of the “don’t eat me” signal CD47, which binds to its receptor SIRPα on macrophages. Targeting of the CD47-SIRPα axis is being tested in numerous cancer types. The cancer cells cultured with MSC^High^ expressed significantly lower levels of CD47 than those cultured with the monocytic cell line (Figure 7B), reinforcing the hypothesis that stromal cells can act on the cancer cells to alter the function and phenotype. Anti-EGFR antibodies are the most common targeted therapy used in the treatment of colon cancer. They act by blocking EGFR signalling, which reduces cell growth and survival signalling and can induce antibody-dependent cellular cytotoxicity [65]. In contrast to PD-L1 and CD47 expression, the induction of EGFR on the cancer cells was significantly increased when cultured with high-density MSCs but was unchanged by the incorporation of the innate immune cell compartment (Figure 7B,C). Taken together, these results further illustrate the complex nature of cellular interactions with the CRC TME and how they can have a significant impact on cancer cell expression of therapeutic targets and immunomodulatory markers.

### 3.8. Stromal Cells Alter Cancer Cell Response to EGFR Inhibition

**Figure 8:**
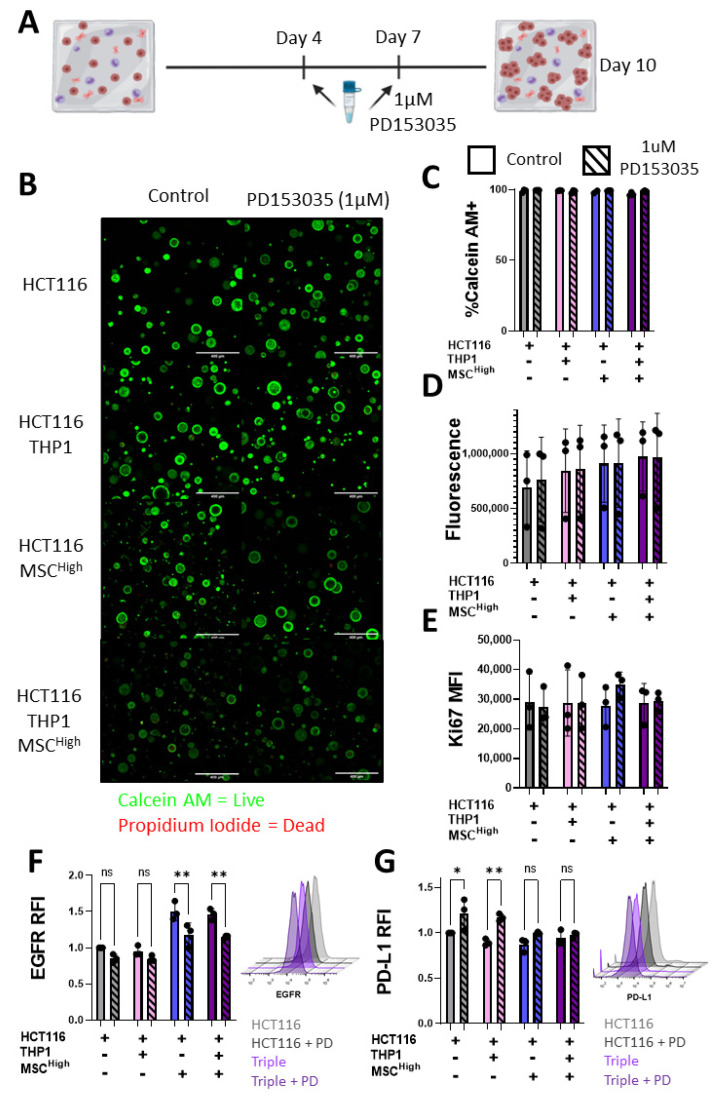
Stromal cells alter CRC response to EGFR inhibitor treatment. (**A**) Schematic description of 10-day GelMA triple culture system, treatment with 1 uM PD153035 at day 4 and day 7. (**B**) Confocal microscopy images of Calcein AM- (live) and Propidium Iodide (dead)-stained gels, scale bar 400 um. (**C**) Quantification of % of Calcein AM-positive or live cells in the gels (*n* = 3 fields within 1 gel). (**D**) Alamar Blue measure of metabolic activity of cell-containing gels at day 10 (*n* = 3). (**E**) Flow cytometry Ki67 expression in EpCAM-positive cells isolated at day 10 (*n* = 3). (**F**) Relative median fluorescent intensity of EGFR on live EpCAM-positive cells isolated from the gels. Representative histograms of the flow cytometer staining, HCT116 (light grey), HCT116 treated with PD153035 (PD) (dark grey), triple culture (light purple) and triple culture treated with PD153035 (dark purple). (**G**) Relative median fluorescent intensity of PD-L1 on live EpCAM-positive cells isolated from the gels. Representative histograms of the flow cytometer staining, HCT116 (light grey), HCT116 treated with PD153035 (PD) (dark grey), triple culture (light purple) and triple culture treated with PD153035 (dark purple). Individual values are displayed as black dots on bar charts. Error bars represent mean ± SD. * *p* < 0.05, ** *p* < 0.01, two-way ANOVA with Šídák’s multiple comparisons test.

EGFR blocking/inhibition is the most successful targeted therapy for the treatment of CRC to date [66]. Our model reveals that EGFR expression is increased on CRC when cultured with stromal cells. To assess the role of the stromal compartment in altering cancer cells’ response to treatment, we used the EGFR tyrosine kinase inhibitor PD153035. GelMA experimental groups were treated at days 4 and 7 with PD153035 (Figure 8A). While PD153035 has been shown to have cytostatic and cytotoxic effects [67,68], live/dead imaging of the spheroids did not display significant cell death following treatment with the EGFR inhibitor at 1 μM (Figure 8B,C). No significant differences in metabolic activity or Ki67 expression were observed between groups (Figure 8D,E). This is likely due to the HCT116 Kras mutant status that promotes survival and cell growth signalling [69,70,71].

While no significant changes in cell death, metabolism or proliferation were observed, there were significant changes in cancer cell surface marker expression when analysed by flow cytometry (Figure 7). Changes between the Triple (MSC^High^) and other cell-containing gels was observed with EGFR and PD-L1 expression (Figure 7B), so we chose to focus on these markers. PD153035 treatment did not alter the cancer cell surface expression of EGFR in the HCTT16 or the HCT116 + THP1 groups (Figure 8F). However, both groups containing MSC^High^ showed a significant reduction of EGFR expression following treatment (Figure 8F). Conversely, only the groups with no MSCs showed altered PD-L1 expression in response to PD153035 treatment (Figure 8G). The HCT116 and HCT116 + THP1 gels showed an increase in PD-L1 expression, but this was not seen in the HCT116 + MSC^High^ or triple culture groups, showing the importance of testing therapies in a representative multicellular construct and emphasising the role of the MSC compartment in shaping the TME.

## 4. Discussion

CRC develops in a complex tumour microenvironment, where different cell types, including tumour cells, fibroblasts, endothelial and immune cells, communicate, which can aid tumour development and alter treatment response. CRC patients that develop tumours with high levels of stromal or immune infiltrate show the lowest disease-free survival rates [13]. However, the role the stromal compartment plays in tumour promotion and response to treatment is not yet fully understood. This may be in part due to the lack of affordable and reproducible in vitro models available for research that combine multiple cell components.

Traditional 2D co-culture systems do not reproduce the complex microenvironment of tumours, lacking cell–cell and cell–matrix interactions, tumour stiffness and extended undisrupted culture times [72]. Animal models are quite expensive and require ethical considerations, and therefore, are not accessible for all researchers [26]. This can contribute to the low efficacy rates when new therapies are brought to clinical trial and may be preventing the identification of novel targets not present in animal models or not observed in 2D single-cell-type cultures. 3D models serve to address the limitations of preclinical models, including reproducibility, sensitivity, cost and ethical considerations that exist in understanding the complexity and cell–cell interactions that dictate the outcome in CRC [72].

To assess the impact of changes in a more physiologically relevant model, we developed a 3D GelMA culture system that enables the assessment of changes influenced by mesenchymal stromal cells and macrophages in CRC. This hydrogel-based 3D culture system facilitates the growth of multiple cell types over the course of 10 days, in an environment where the stiffness is modifiable. Well-formed spheroids were produced with features reminiscent of in vivo tissue orientation and facilitated signalling between the cells in the hydrogel. 

While this model is less complex than some other 3D models, such as microfluidic devices, it has the benefit of being easy to use, reproducible, sensitive and affordable, which makes it a more accessible, higher throughput option for large drug testing screens [73]. The protocol for generating GelMA is outlined extensively by Loessner et al. and can be established in-house using commonly available equipment, over 1–2 weeks [32]. The lyophilisation of the GelMA allows it to be stored in a stable manner for extended periods of time. This enables batch making of the GelMA to support a large number of experiments from one large production. Making the cell-containing GelMA hydrogels requires a UV cross-linker and reusable moulds, and the drawings for the reusable moulds are outlined by Loessner et al. and can be customised [32]. This makes the setup of this 3D culture system relatively inexpensive and customisable compared to more complex culture systems. The hydrogel and moulds are easy to handle, can be prepared by a single user and do not require extensive training, which is necessary with many more complex systems. These practical considerations paired with the biocompatibility, sensitivity and ability to modify the stiffness of the GelMA hydrogels makes this a strong choice for use in biological 3D model development.

Similar to observations in human CRC datasets, our novel triple culture GelMA model identified that incorporating stromal cells into a 3D cancer cell culture system can alter the transcription of ECM proteins and matrix remodelling enzymes. These findings further support the use of our model, as fibronectin has been implicated in CRC proliferation, migration, metastasis and chemo-resistance [59,74,75]. The ECM in CRC is altered through the stages of development with an increase in the stiffness of the tissue observed with increasing disease burden [52,76]. MMPs are enzymes that degrade and remodel the ECM and have been linked to cancer cell invasion, metastasis and angiogenesis, with MMP-1, -2, -7, -9 and -13 expression shown to correlate with worse outcomes [44,77,78]. Both MSCs and innate immune cells have been proven to contribute to the formation of the ECM in the TME [79,80]. The incorporation of stromal cells enhanced ECM remodelling transcripts of molecules often seen to be elevated in patient samples, indicating the importance of relevant culture models. This work also supports the use of this model for developing and testing therapeutics that target invasion and metastasis in colon cancer. Using patient-specific cellular components in the GelMA model may enable sensitive identification of dysregulated cell–ECM and/or cell–cell interactions that could represent novel targets or inform personalised therapies.

The impact of the immune system on tumour progression is well-accepted. In fact, Hanahan and Weinberg outlined in their Hallmarks of Cancer: Next Generation, that for a tumour to be successful, it must be capable of avoiding immune destruction and develop in a tumour-promoting inflammatory microenvironment [81]. Cells of the monocyte lineage play a pivotal role in the development of the tumour-promoting inflammation, with macrophages being a common component of the colon TME [82,83,84,85]. We have shown that incorporation of stromal cells into a 3D culture system can significantly increase the secretion of cytokines and chemokines that induce macrophage recruitment and function and contribute to the tumour-promoting inflammation. IL-8 exerts tumour-promoting effects including EMT in the TME. It signals through the CXCR2/CXCR1 receptors and targeting CXCR2/CXCR1 caused repression of tumour growth in lung and melanoma models [86,87]. Our observations that CXCL1 and IL-8 are enhanced by the inclusion of stromal cells in 3D cultures of CRC cells and macrophages indicate that targeting the CXCR1/2 signalling axis may represent a therapeutic target in stromal-dense CRC tumours. As well as its role in chemotaxis of neutrophils and macrophages, CXCL12 secretion from stromal cells has been shown to polarise macrophages to IL-10-expressing macrophages in the colon, highlighting an important stromal cell macrophage-signalling axis that regulates inflammation [88]. CXCL12 suppresses the anti-tumour functions in macrophages in the TME [89]. Whether the stromal cells express these chemokines or cytokines in the TME, or induce expression in cancer cells or macrophages, is unclear. However, understanding cell-specific regulation of these factors in the CRC TME may offer novel insights into potential targeting approaches. Targeting this immunosuppressive environment and re-engaging the anti-tumour effector functions of macrophages has become the aim of many new therapies for the treatment of colorectal cancer. GelMA-based culture systems represent a sensitive model in which immunotherapeutics impact on macrophage function may be determined. 

Immunotherapies are becoming a mainstay therapy in many advanced and difficult to treat cancers. They have the potential to initiate unique immunological memory compared with other therapies making them useful for the treatment in both metastatic and primary disease [90]. While these novel treatments provide the hope of life-long protection, a response is only seen in about 20% of patients treated with immunotherapeutics [91,92]. The key to understanding the lack of efficacy lies in a more comprehensive knowledge of cellular interactions in the TME. The addition of stromal cells to the 3D culture system altered the CRC expression of EGFR, CD47 and PD-L1, clinically assessed monoclonal antibody targets in CRC. Better understanding of the expression of these therapeutically targeted molecules could reveal novel interventions and support the stratification of patients to improve treatment response rates.

EGFR blockers/inhibitors are the most clinically used targeted therapy for the treatment of CRC. Using PD153035, an EGFR tyrosine kinase inhibitor, we demonstrated that targeting EGFR did not affect the proliferation or cell death of cancer cells in the 3D system. However, the expression of EGFR was significantly reduced on cancer cells co-cultured with MSCs, and PD-L1 was increased on cancer cells not cultured with MSCs following treatment. It has been shown that PD-L1 induction can result from cell stress, acting as a protective measure [93,94], suggesting that the MSCs may be acting to reduce cancer cell stress in response to the EGFR inhibitor, and further emphasising the importance of including key cellular components of the TME in in vitro studies.

This work reinforces the importance of using relevant in vitro models and the potential to expand our knowledge of cellular interactions in the complex CRC TME through this 3D model system. With this knowledge, patients could be better stratified for treatment response and new therapeutic targets may be identified.

## 5. Conclusions

The stromal compartment of the CRC TME contributes to ECM formation, ECM remodelling, immune suppression and the induction and maintenance of an inflammatory microenvironment. These factors can alter tumour growth, metastasis, response to treatments and patient survival rates. Having accessible, reproducible models that allow the controlled modulation of each cell type is vital for the identification and testing of novel therapeutic targets and for gaining a better understanding of cellular interactions in CRC, which remains the third leading cause of cancer-related deaths worldwide. Here, we have developed a 3D triple culture model of CRC combining cancer cells, MSCs and monocytes in a tuneable hydrogel that can be analysed using a variety of techniques to assess cellular phenotype, transcriptional profiles, surface and secreted proteins. We have shown that this multicellular GelMA model has huge potential for screening targeted immunotherapeutics for CRC, due to its ease of use, reproducibility and cost effectiveness.

## Figures and Tables

**Table 1:** Probes for RT-qPCR.

Target	Assay ID	Label	Supplier/Cat#
Beta-2-Microglobulin	Hs00187842_m1	VIC-MGB	ThermoFisher Scientific 4448489
Fibronectin 1	Hs01549976_m1	FAM-MGB	ThermoFisher Scientific 4331182
Collagen 1A1	Hs00164004_m1	FAM-MGB	ThermoFisher Scientific 4331182
Versican	Hs00171642_m1	FAM-MGB	ThermoFisher Scientific 4331182
MMP2	Hs01548727_m1	FAM-MGB	ThermoFisher Scientific 4331182
MMP9	Hs00957562_m1	FAM-MGB	ThermoFisher Scientific 4331182

**Table 2:** Antibodies and viability dyes for flow cytometry.

Antigen/Product	Clone	Catalogue No.	Supplier
EpCAM	9C4	324204	BioLegend, San Diego, CA, USA
PD-L1	29E.2A3	329706	BioLegend San Diego, CA, USA
CD47	CC2C6	323114	BioLegend San Diego, CA, USA
EGFR	AY13	352920	BioLegend San Diego, CA, USA
Ki67	11F6	151210	BioLegend San Diego, CA, USA
Zombie Violet™ Fixable Viability Kit		423114	BioLegend San Diego, CA, USA
SYTOX™ Blue Dead Cell Stain		S34857	ThermoFisher Scientific Waltham, MA, USA

## Data Availability

Publicly available datasets were analysed in this study. This data can be found at Geo2R: https://www.ncbi.nlm.nih.gov/geo/geo2r/, accessed on 20 September 2021, GSE39397, GSE35602. The data presented in this study are available upon request from the corresponding author.

## Supplementary Materials

The following are available online at https://www.mdpi.com/article/10.3390/cancers13235998/s1, Supplemental Figure S1. TEM images of spheroids show cancer cells appear to regain their epithelial like structure in 3D.
