# Supplementary material for: Stromal Cells Promote Matrix Deposition, Remodelling and an Immunosuppressive Tumour Microenvironment in a 3D Model of Colon Cancer
Source: Cancers (Basel). 2021 Nov 29;13(23):5998. doi: 10.3390/cancers13235998 (PMC8656544; doi:10.3390/cancers13235998)
Supplement: Supplementary file 1 [file cancers-13-05998-s001.zip › cancers-1409159-supplementary.pdf]

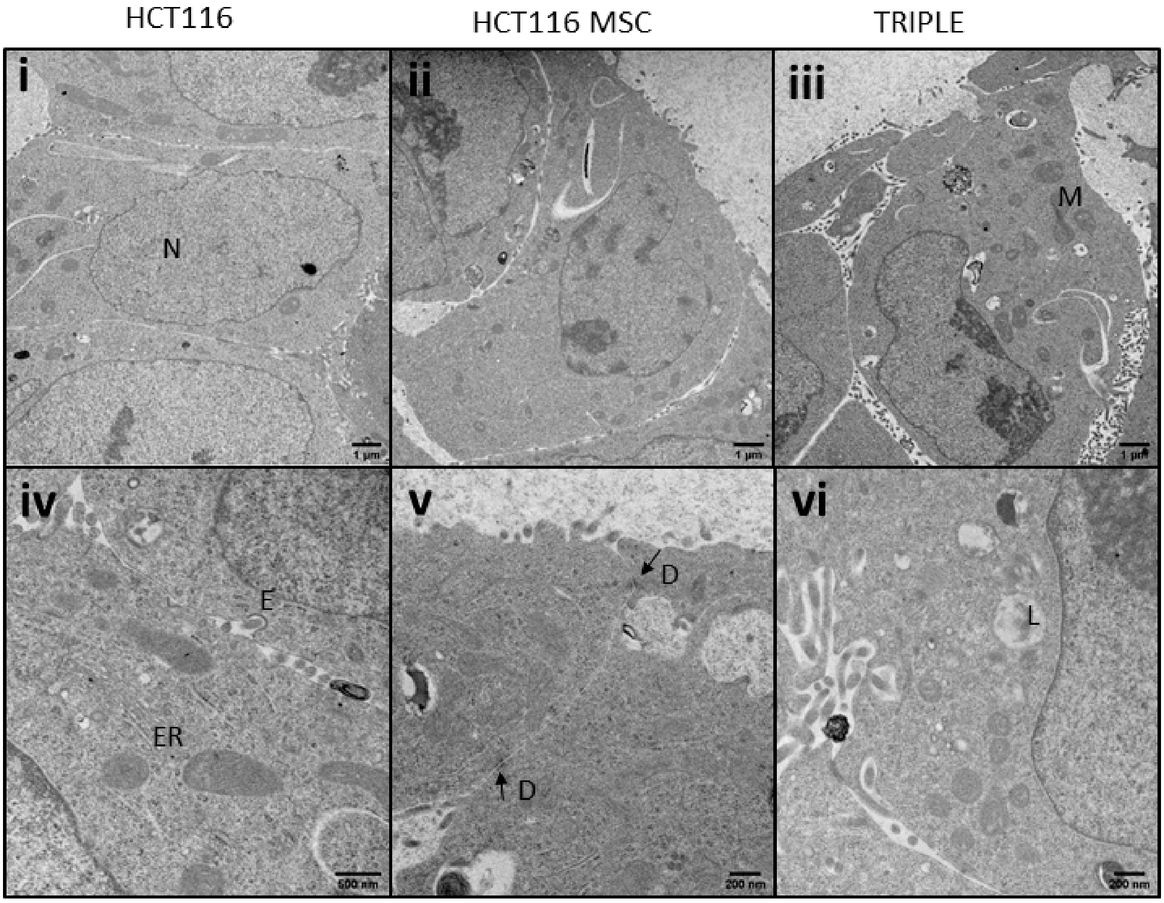

Supplementary Figure S1. TEM images of spheroids show cancer cells appear to regain their epithelial like structure in 3D. Cells display high levels of mitochondria. Transmission electron microscopy images display cellular organisation in the spheroids, HCT116 gels (**i,iv**), HCT116 MSC (**ii,v**), triple culture (**iii,vi**). Organelles including nuclei (N), mitochondria (M), endoplasmic reticulum (ER), endo/exocytotic vesicle (E), desmosome (D) and autolysosome (L) are visible. Scale = 1 µm (**i,ii,iii**), 600 nm (iv), 200 nm (**v,vi**).
